# Inducing apoptosis using chemical treatment and acidic pH, and detecting it using the Annexin V flow cytometric assay

**DOI:** 10.1371/journal.pone.0270599

**Published:** 2022-06-29

**Authors:** Catherine M. Worsley, Rob B. Veale, Elizabeth S. Mayne

**Affiliations:** 1 Department of Immunology, Faculty of Health Science, University of Pretoria, Pretoria, South Africa; 2 Department of Molecular Medicine and Haematology, Faculty of Health Sciences, University of the Witwatersrand, Johannesburg, South Africa; 3 National Health Laboratory Service, Johannesburg, South Africa; 4 School of Molecular and Cell Biology, Faculty of Science, University of the Witwatersrand, Johannesburg, South Africa; 5 Department of Immunology, Faculty of Health Sciences, University of the Witwatersrand, Johannesburg, South Africa; 6 Division of Immunology, Department of Pathology, Faculty of Health Sciences, University of Cape Town, Cape Town, South Africa; Ege University Faculty of Medicine: Ege Universitesi Tip Fakultesi, TURKEY

## Abstract

Cell death is important in physiology, and can happen as a result of structural damage, or as a sequence of programmed cellular processes known as apoptosis. Pathogenic alterations in apoptosis occur in a number of diseases, including cancer, viral infections, autoimmune diseases, immunodeficiencies, and degenerative conditions. Developing accurate and reproducible laboratory methods for inducing and detecting apoptosis is vital for research into these conditions. A number of methods are employed to detect cell death, including DNA fragmentation, the TUNEL assay, and electron microscopy although each has its limitations. Flow cytometry allows for the distinction between live, early apoptotic, late apoptotic and necrotic cells. In this protocol we successfully induce apoptosis using chemical treatment and treatment with low pH in solid tumour cell lines, and have optimized detection using the Annexin V/PI apoptosis assay.

## Introduction

The importance of studying death in cell culture is becoming increasingly apparent. Cell death is a key component of physiology, and can happen either passively as a result of structural damage (necrosis), or actively as a sequence of programmed cellular processes such as apoptosis. Apoptosis or programmed cell death (PCD) is a genetically organized process that occurs when survival and proliferative signals are removed, or when the cell has suffered DNA damage [1]. Cells undergoing PCD are broken down into membrane-bound apoptotic bodies or vesicles which are taken up by neighbouring cells and phagocytes [1]. These apoptotic vesicles aid cell-to-cell communication, and are able to transmit nucleic acid, proteins, and lipids to other cells, aiding in both wound healing and cell survival [2, 3]. In contrast, necrosis is characterized by swelling of the cell and mitochondria, and cell membrane rupture with release of inflammatory cellular contents into the surrounding microenvironment [1]. Aberrations in PCD are pathogenic in many processes. Reduced PCD is associated with malignancies, persistent viral infections, or autoimmune disease, whereas excessive PCD may cause immunodeficiency or degenerative disease [4].

The role that PCD plays in cancer progression is controversial. Apoptosis can promote cancer, and dying cells can affect their environment by stimulating the proliferation of neighbouring cells, affecting intra-tumoural cell competition, and exerting paracrine effects on the tumour microenvironment (TME) [5]. High levels of apoptosis in some tumours correlate with poor prognosis [5]. Prostaglandin E2 (PGE2) is released by apoptotic cells, and has pleiotropic effects on surrounding cells, including proliferative and immune evasive effects [5]. Other studies however show that cancer cells block apoptosis by preventing p53 signalling, overexpressing antagonists to apoptotic pathways, immune evasion, and inducing apoptosis of lymphocytes via programmed death ligand-1 (PD-L1) [6]. As cell death contributes to a number of adverse processes, understanding how it is regulated may provide insight into disease pathology.

### 1. Inducing apoptosis in the laboratory

Any environmental agent or condition that stresses the cell can cause cell death. In cell culture systems, the severity and type of cell death can be controlled. Death can be induced by heat [7], ultra-violet (UV) light [7, 8], various chemicals [7, 9], production of reactive oxygen species (ROS) [7, 10, 11], nutrient or growth factor deprivation [10–12], Fas introduction [8], cytokines [11, 13], viruses [12], and acidosis [10, 11]. Cultured cells studied in isolation from their normal physiological surroundings often behave differently than *in vivo* as they are unable to communicate with neighbouring cells, and elements of their microenvironment. Our aim was to develop a robust method of inducing apoptosis in cultured cells that would mimic their natural microenvironment. As the tumour microenvironment is typically more acidic [14], we used culture media of decreasing pH to induce cell death and compared this to cell death induced by chemicals such as dimethyl sulfoxide (DMSO) and stauroporine (STS) which have been well reported [9, 11, 15, 16].

### 2. Confirming apoptosis in the laboratory

A number of techniques can be used to detect apoptosis. Some physical features of PCD can be observed by microscopy [17]. Cells undergoing PCD have distinct morphological features including cell shrinkage, plasma membrane blebbing, with nuclear condensation and DNA fragmentation [4]. Other techniques to study PCD include observing DNA fragmentation, detecting changes in caspase and mitochondrial activity, or changes in phosphatidylserine (PS) detection. These techniques all have their positive and negative attributes which have been well described elsewhere [12, 17, 18] and are summarized in Table 1.

**Table 1:** Comparison of apoptosis assays.

Assay	Apoptotic characteristic measured	Benefits	Limitations
DNA laddering	DNA fragmentation by endonucleases	Inexpensive	Only detects late apoptosis
			Needs apoptosis in a large number of cells to be detects
TUNEL (Terminal dUTP Nick-End Labeling)	3’end labelling of DNA fragments	Commercial kits available	High cost
			Fixation and pretreatment affect DNA strand breaks
			Doesn’t distinguish between apoptosis and necrosis
Caspase-3 Activity	Measure activity of caspase-3 which is activated in the terminal apoptosis cascade	Quantitative	Integrity of tissue destroyed
			Lots of tissue needed to detect activity
			Can’t determine which cell is apoptotic
Mitochondrial assays	Mitochondrial redox status, Ca^2+^ increase, reactive oxygen species (ROS), mitochondrial permeability, mitochondrial depolarization	Assayed in living cells	Difficult to distinguish between apoptosis and necrosis
Viability dyes	Bind to exposed nucleic acids	Fast	Unable to distinguish between apoptosis and necrosis
		Inexpensive	Cannot penetrate thick tissue
Microscopy (TEM or fluorescent)	Morphological changes including membrane blebbing, cell swelling, and nuclear condensation	Structural characteristics of apoptosis observed	Time-consuming
			Limited samples can be analysed
Annexin V assay	Phosphatidylserine exposure on plasma membrane	Detects early apoptosis	Expensive in whole animal studies
		Can be combined with other multiple labels of other proteins or DNA	
		Quantitative	

Flow cytometry has many advantages over other techniques, as it is rapid, and can be performed on relatively simple analysers. The technique is quantitative, and analysis of cell death on individual cells or cell populations can be performed [19]. Cells undergoing PCD can be distinguished from viable cells based on their light scatter properties. In early apoptosis, cell shrinkage occurs while the cell membrane remains intact, causing a decrease in cell size measured by forward scatter (FSC) while cell composition, measured by side scatter (SSC), remains the same. As PCD progresses, changes in both FSC and SSC occur. As the cell membrane becomes more permeable, the cell shrinks causing a decrease in cell size. Nuclear degranulation increases side scatter [4]. During necrosis however, the cytoplasm swells causing an increase in cell size and FSC, while release of intracellular contents causes a decrease in SSC [4]. FSC and SSC alterations alone are however insufficient in distinguishing between apoptotic and necrotic cells.

**Fig 1:**
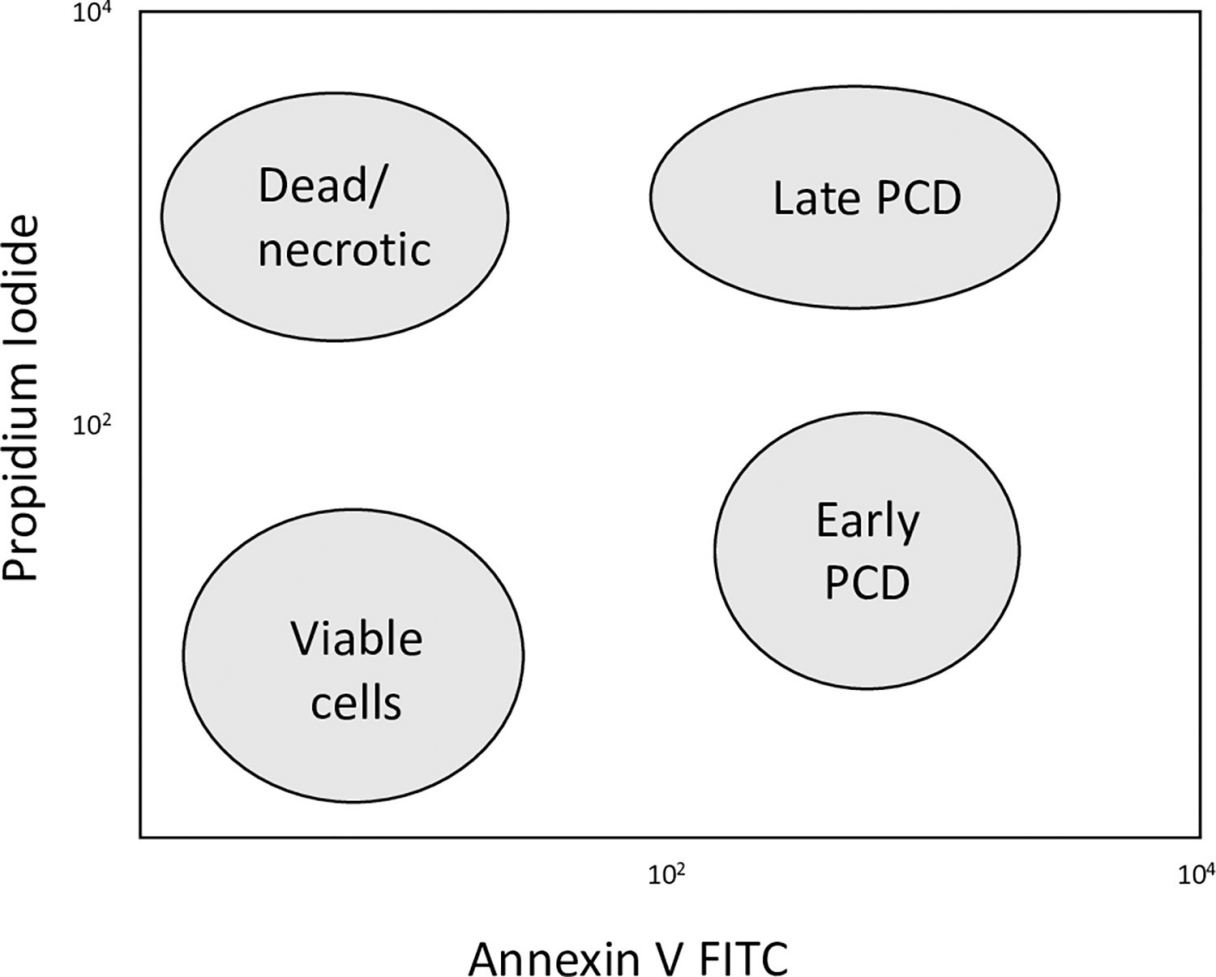
An example of a flow cytometry plot showing stages of cell death. Viable cells do not bind Annexin V or take up PI. Cells in early PCD bind Annexin V but still exclude PI. As the cell membrane becomes more permeable, cells in late PCD bind to Annexin V and PI. Dead or necrotic cells do not bind to Annexin V, but do take up PI.

Based on the benefits of being able to distinguish between early and late PCD, we used the Annexin V assay performed by flow cytometric methods (FCM). In early PCD, although the plasma membrane is intact, PS begins to be redistributed from the cytoplasmic surface to the exterior of the plasma membrane. As PCD progresses, PS is completely exposed and the plasma membrane becomes more permeable. Annexin V binds to PS and is labelled with a fluorophore that is detected by a flow cytometer [20]. As a number of markers can be used simultaneously in flow cytometry, multiple cell death characteristics in different cell populations can be detected at the same time. FCM also distinguishes between live and dead cells by measuring the uptake of viability dyes such as propidium iodide (PI). The PI intercalates with exposed DNA, and the fluorescence detected indicates how much dye is bound. As the plasma membrane is intact in viable cells and those undergoing early PCD, the PI is excluded. As the plasma membrane becomes more permeable as PCD progresses, more and more dye is able to enter the cell [19]. Viability dyes alone are, however, inadequate to distinguish different stages of PCD. By using Annexin V and PI in one assay, we were able to distinguish between different stages of cell death, including viable, early PCD, late PCD and dead or necrotic cell populations (Fig 1).

## Materials and methods

The protocol described in this article is published on protocols.io dx.doi.org/10.17504/protocols.io.b2dfqa3n and is included for printing as S1 File with this article.

This study was conducted on long-established cell lines, and no additional patient consent was needed. This study was approved by Human Research Ethics Committee (HREC) of the University of the Witwatersrand (approval M120205).

### Expected results

The assay has been optimised in both breast (MCF-7) and oesophageal squamous cell carcinoma cell lines (SNO). Cells were treated with DMSO and STS, and cell death was measured by flow cytometry. We monitored morphological changes of the cells treated with DMSO and STS under the microscope every 15 minutes. At 30 minutes, no morphological change was observed. At 1 hour, some cells were starting to round up, and at 2.5 hours, some cells had detached and were floating in the medium.

**Fig 2:**
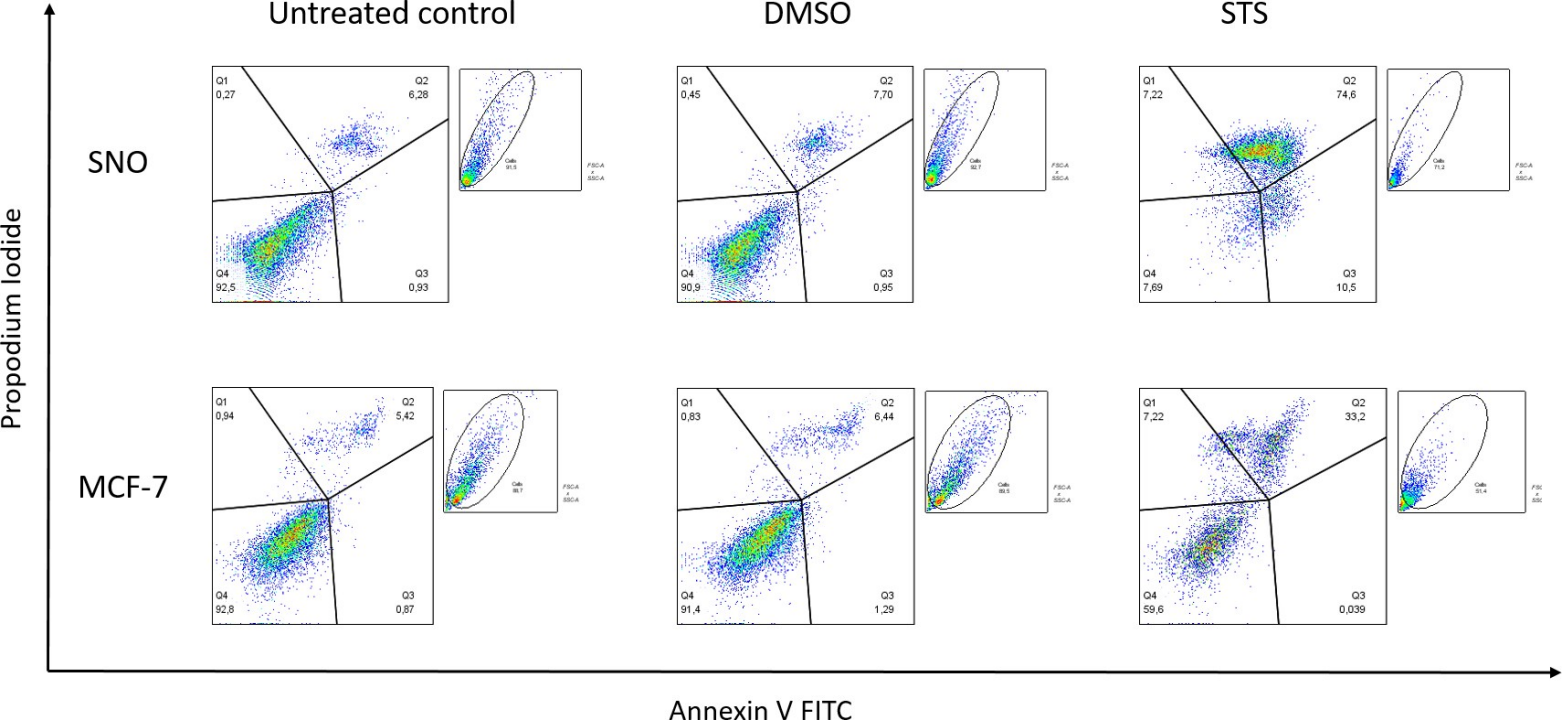
Induction of programmed cell death in an MCF-7 breast carcinoma and SNO oesophageal squamous cell carcinoma cell lines.

An example of PCD induction in the MCF-7 and SNO cell lines is given in Fig 2. An untreated control should always be included, as the death detected here can be attributed to the harvesting of the cells with enzymatic treatment with TE buffer, and death that may have occurred during centrifugation and assay preparation. Differences in cell death are noticeable between treated and untreated cells, as DMSO and STS exhibited much higher levels of cell death than the untreated control. We observed that cells treated with STS became smaller and less complex as death was induced. STS caused more cell death than DMSO.

**Fig 3:**
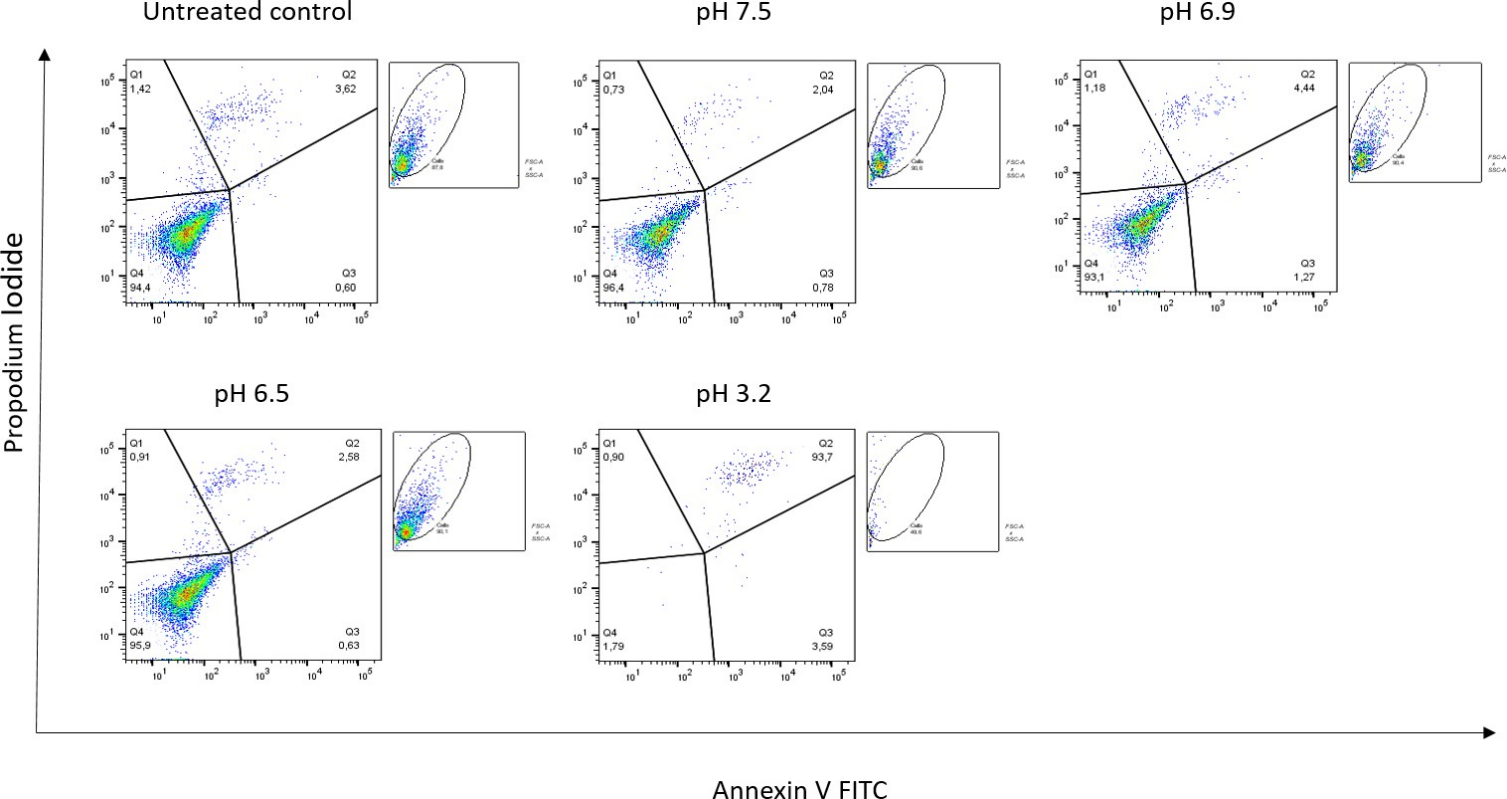
Decreasing pH causes cell death in SNO cell line.

In order to determine the effects of pH, SNO cells were treated with media of decreasing pH (pH 7.5, 6.9, 6.5, 3.2) (Fig 3). After 6 hours, we performed the FCM assay, and measured PCD of the different treatments. Cells treated with media of pH 7.5 appeared very much like the untreated control. As pH decreased to 6.5 however, more cells were detected in early and particularly late PCD. As the media became more acidic, cells tend to follow a more necrotic form of cell death. We suggest that researchers try the assay with media of varying pH levels to determine the correct pH for their work. Time of treatment also needs to be carefully monitored in order to detect cells still in early PCD.

## Conclusion

We successfully optimised induction of cell death in solid tumour cell lines. Lowering pH mimics the acidic TME, and as pH decreases PCD is induced. The flow cytometric Annexin V/PI assay is a useful tool in distinguishing between viable cells, and those in early and late PCD, and can be combined with other markers to characterize PCD in different cell populations. This protocol will assist researchers in studying PCD in a range of tumour cell lines.

## Supporting information

S1 File Loaded on protocols.io dx.doi.org/10.17504/protocols.io.b2dfqa3n. (PDF)
