# Supplementary material for: Inducing apoptosis using chemical treatment and acidic pH, and detecting it using the Annexin V flow cytometric assay
Source: PLoS One. 2022 Jun 29;17(6):e0270599. doi: 10.1371/journal.pone.0270599 (PMC9242499; doi:10.1371/journal.pone.0270599)
Supplement: S1 File — (PDF) [file pone.0270599.s001.pdf]

b2p9qdr6

Inducing apoptosis using chemical treatment and acidic pH, and detecting it using the Annexin V flow cytometric assay V.(b2p9qdr6)

Catherine M. Worsley[1,2,3], Rob B. Veale[4], Elizabeth S. Mayne[5,3]
[1]Department of Molecular Medicine and Haematology, Faculty of Health Sciences, University of the Witwatersrand;
[2]Department of Immunology, Faculty of Health Science, University of Pretoria;
[3]National Health Laboratory Service;
[4]School of Molecular and Cell Biology, Faculty of Science, University of the Witwatersrand;
[5]Department of Immunology, Faculty of Health Sciences, University of the Witwatersrand

1

worsleycm

Cell death is important in physiology, and can happen as a result of structural damage, or as a sequence of programmed cellular processes known as apoptosis. Pathogenic alterations in apoptosis occur in a number of diseases, including cancer, viral infections, autoimmune diseases, immunodeficiencies, and degenerative conditions. Developing accurate and reproducible laboratory methods for inducing and detecting apoptosis is vital for research into these conditions. A number of methods are employed to detect cell death, including DNA fragmentation, the TUNEL assay, and electron microscopy although each has its limitations. Flow cytometry allows for the distinction between live, early apoptotic, late apoptotic and necrotic cells. In this protocol we successfully induce apoptosis using chemical treatment and treatment with low pH in solid tumour cell lines, and have optimized detection using the Annexin V/PI apoptosis assay.

Catherine M. Worsley, Rob B. Veale, Elizabeth S. Mayne . Inducing apoptosis using chemical treatment and acidic pH, and detecting it using the Annexin V flow cytometric assay. **protocols.io**
https://protocols.io/view/inducing-apoptosis-using-chemical-treatment-and-ac-b2p9qdr6

National Research Foundation
Grant ID: TTK20110727000022309

Dec 08, 2021

Dec 08, 2021

55777

Annexin V FITC Apoptosis Detection kit **BD Biosciences Catalog #BD/556570**

Cytometer Setup and Tracking beads **BD Biosciences Catalog #BD/641319**

Dimethyl sulfoxide (DMSO) **Sigma – Aldrich Catalog #D8418**

Dulbeccos Modified Eagles Medium (DMEM) **Sigma Aldrich Catalog #D5030**

FACSFlow Sheath fluid **BD Biosciences Catalog #BD/342003**

Fetal Bovine Serum **Sigma – Aldrich Catalog #F2442**

Hams F12 **Sigma – Aldrich Catalog #N6658**

Hydrochloric acid **Sigma – Aldrich Catalog #H1758**

Phosphate Buffered Saline (PBS) **Merck Millipore Catalog #524650**

Penicillin-Streptomycin **Sigma – Aldrich Catalog #P4333**

Staurosporine (STS) **Immunochemistry Technologies Catalog #6212**

Trypan blue **Sigma – Aldrich Catalog #T8154**

Trypsin EDTA **Merck Millipore Catalog #SM-2004-C**

LSR II
Flow Cytometer
BD 0000

Cell culture of mammalian solid tumour cells lines

Bring all reagents to 37°C in a waterbath.

1 Note: ensure that all surfaces and equipment are cleaned with 70% ethanol. Perform all work in a laminar airflow cabinet.

2 In a 10cm culture dish, add 10ml of Dulbecco's Modified Eagle's Medium (DMEM)/Hams F12 (3:1) supplemented with 10% fetal calf serum (FCS) and 2% penicillin/streptomycin.

3 Add the cell suspension to the dish, then incubate at 37°C with humidity, and 5%C CO_2 in air to mimic *in vivo* conditions.

Cell detachment

4 Bring all reagents to 37°C in a waterbath.

5 Observe cell growth daily using an inverted light microscope

6 When cells reach 80% confluency, remove the culture media and wash the cells with 2ml phosphate buffered saline (PBS). Discard the PBS.

7 Add 2ml of trypsin/EDTA (TE) solution and incubate at 37°C for 5-10 mins or until cells start to detach from the culture dish. Note: do not leave cells in TE for too long as it can cause damage to the cells. To speed up detachment, lightly move the dish so that the TE covers the whole area.

Cell counting using Trypan Blue

8 Once cells are detached, add 2ml of the cell-TE mixture to 8ml of DMEM/HAMS F12 medium.

9 On a piece of parafilm, pipette 20µl of 0.4% trypan blue.

10 Add 20µl of the cell mixture to the trypan blue on the parafilm, and gently mix together by slowly pipetting up and down

11 Add 20µl of the cell/trypan blue mixture to the chamber of the haemocytometer, and cover with a coverslip. This will ensure that the liquid moves onto the counting chambers by capillary action. Ensure that there are no bubbles under the coverslip.

12 Focus the microscope on the gridlines using the 10x objective.

13 Count the lives cells (live cells exclude trypan blue) in one set of 16 squares.

14 Move the haemocytometer to count the cells in the next set of 16 squares. Do this 4 times.

15 1. To calculate the number of viable cells/ml:
a.Add the total number of cells together
b.Divide by the number of sets of squares counted to get the average number of cells.
c.Multiply by the dilution factor (2)
d.Multiply by 10 000 (10^4) (this is the volume of the squares)
e.Calculate the volume needed to get the required number of cells per dish (in this case 150 000)
Example:
<u>Number of cells (84)</u>
Number of block sets (4)= 21 (average number of cells per block)

21 x dilution factor (2) x 10 000 = 420 000 cells/ml

<u>Number of cells wanted (150 000)</u>
Cells/ml (420 000)= 0.357ml of cell suspension to each dish

16 Pipette the volume of cell suspension calculated above into the number of 6cm dishes required (7 dishes).

17 Add culture medium to the dish to make the total volume up to 1ml

18 Place dishes into the incubator and leave overnight at 37°C with humidity, with 5% CO_2 in air.

Cell death induction

19 Prepare the Staurosporine (STS) and pH-adjusted media.

20 STS preparation:
a.Dissolve STS powder in tissue culture grade DMSO to obtain a 1mM concentration

21 pH-adjusted media preparation:
Adjust medium pH to required pH using hydrochloric acid (HCl) and measure with a pH meter

22 Remove all the dishes from the incubator, and wash the cells with 2ml of PBS.

23 Treat the 7 dishes as follows:

Dishes	Dish 1 - untreated control	Dish 2 - DMSO	Dish 3 - STS+DMSO	Dish 4 - pH 6.5 15min	Dish 5 – pH 6.5 1hr	Dish 6 – pH 3.2 15min	Dish 7 – pH 3.2 1hr
Initial steps	Add 6ml of serum-free DMEM/Hams F12 medium	Add 10µl DMSO per ml of culture media (i.e. 60µl DMSO in 6ml culture media)	Add 10µl STS+DMSO per ml of culture media	Add 6ml of pH 6.5 media. Place back into the incubator for 15 minutes.	Add 6ml of pH 6.5 media. Place back into the incubator for 1 hour.	Add 6ml of pH 3.2 media. Place back into the incubator for 15 minutes.	Add 6ml of pH 3.2 media. Place back into the incubator for 1 hour.
Additional steps	None	None	None	Remove media after 15min and wash cells in 2ml PBS. Remove PBS and add 6ml serum-free media	Remove media after 1 hr and wash cells in 2ml PBS. Remove PBS and add 6ml serum-free media	Remove media after 15min and wash cells in 2ml PBS. Remove PBS and add 6ml serum-free media	Remove media after 1 hr and wash cells in 2ml PBS. Remove PBS and add 6ml serum-free media
Incubation	Incubate overnight at 37°C						

Annexin V FITC and Propidium Iodide staining

24 Remove medium (with floating cells) and place in a 15ml tube. Centrifuge at 1000rpm for 5 min.

25 Remove the conditioned media supernatant and store at -20°C.

26 Trypsinize adherent cells in 1 ml TE and add to a tube containing 4ml of DMEM/HAMS F12 culture media.

27 Centrifuge at 1000rpm for 5 min. Discard the supernatant.

28 Resuspend pellets of both tubes in PBS and combine into 1 tube.

29 Add 5ml of PBS. Centrifuge at 1000rpm for 5 min. Discard supernatant.

30 Resuspend cells in 1-2ml of 1x binding buffer

31 To 100µl of cell suspension, add 5µl of Annexin V FITC and 5 µl of propidium iodide (PI)

32 Incubate at room temperature in the dark for 15 min

33 Add 200 µl of binding buffer and acquire on the BD LSR II flow cytometer.

Flow Cytometer setup

34 Switch the BD LSR II flow cytometer on and open FACSDiva software. Allow the instrument to

warm up for at least 30 minutes. Ensure that maintenance has been performed and that the instrument is in good working order.

35 Verify instrument performance by running BD Cytometer Setup and Tracking (CST) beads

36 Create an experiment in FACSDiva software

37 To adjust photon multiplier tube (PMT) voltages for compensation, prepare unstained and single stained tubes as follows:
a. Tube 1 - 100µl of unstained cells
b. Tube 2 - 100µl of cells stained with 5µl Annexin V FITC
c. Tube 3 - 100µl of cells stained with 5µl PI

38 Acquire unstained cells. Adjust PMT voltages to ensure that cells are in the negative quadrant for all fluorophores
Note: this needs to be done as some cells autofluoresce which can interfere with the assay

39 Acquire tube 2. Adjust PMT voltages so that there is a well separated positive and negative population in the FITC channel. Adjust PMT voltages so that all other channels only have a negative population.

40 Acquire tube 3. Adjust PMT voltages so that there is a well separated positive and negative population in the PerCP-Cy5.5 channel. Adjust PMT voltages so that all other channels only have a negative population

41 Once compensation has been set up, acquire experimental tubes
Note: acquire and record as many cells as possible for analysis.

42 Perform maintenance and shutdown of the flow cytometer as per manufacturer's instructions

Analysis

43 Export the FCS data files in FSC 2.0 and 3.0 to a folder or external harddrive

44 Import FSC files into FlowJo software

45 Select the untreated control tube and double click. A forward scatter (FSC) vs. side scatter (SSC) plot should open

46 1. Select the cell population of interest and using the gating tool to draw a gate around the population. Name or label that population of cells.

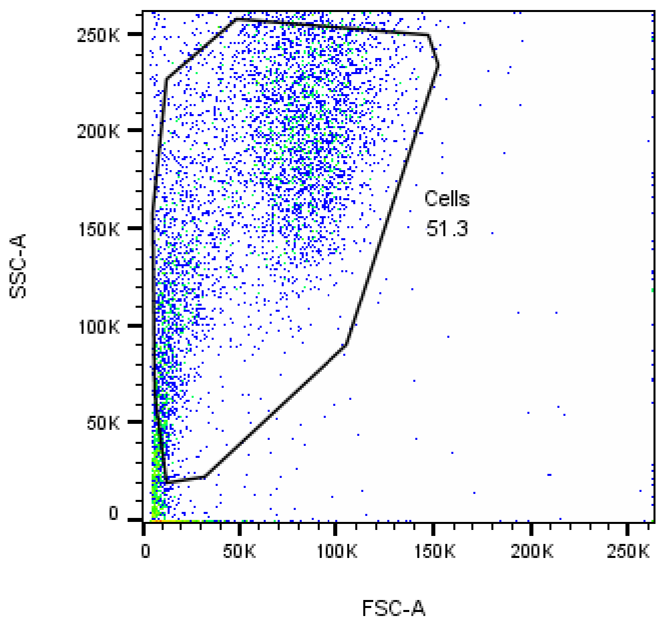

Figure 1. FSC vs. SSC plot

47 Double click on that gate and another FSC/SSC plot should appear.

48 Click on the X–axis and change it to Annexin V or the FITC channel

49 Click on the Y-axis and change it to the PI or PerCP-Cy5.5 channel

50 1. Insert a quadrant or spider quadrant gate as shown below in Figure 2. Gates should separate live cells (Q4: Annexin V-/PI-), early PCD (Q3: Annexin V+/PI-), late PCD (Q2: Annexin V+/PI+) and dead/necrotic (Q1: Annexin V-/PI+) from each other. The numbers in the quadrants indicate the percentage of cells in that quadrant

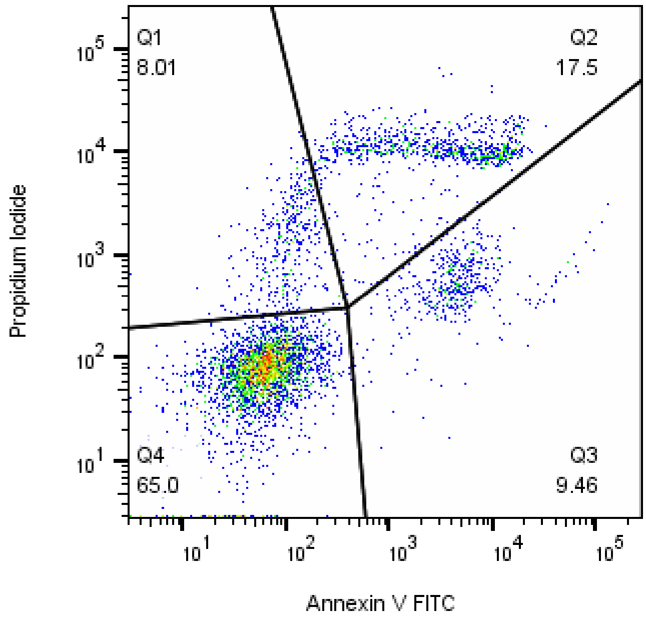

Figure 2. Annexin V FITC vs. Propidium Iodide showing cells undergoing PCD
